# Integration of ATAC-seq and RNA-seq analysis identifies key genes affecting intramuscular fat content in pigs

**DOI:** 10.3389/fnut.2022.1016956

**Published:** 2022-10-05

**Authors:** Zhong Xu, Junjing Wu, Jiawei Zhou, Yu Zhang, Mu Qiao, Hua Sun, Zipeng Li, Lianghua Li, Nanqi Chen, Favour Oluwapelumi Oyelami, Xianwen Peng, Shuqi Mei

**Affiliations:** ^1^Hubei Key Laboratory of Animal Embryo and Molecular Breeding, Institute of Animal Husbandry and Veterinary, Hubei Provincial Academy of Agricultural Sciences, Wuhan, China; ^2^The John Curtin School of Medical Research, Australian National University, Canberra, ACT, Australia

**Keywords:** pig, ATAC-seq, RNA-seq, transcription factor, chromatin accessibility

## Abstract

Meat quality is one of the most important economic traits in pig breeding and production, and intramuscular fat (IMF) content is the major factor in improving meat quality. The IMF deposition in pigs is influenced by transcriptional regulation, which is dependent on chromatin accessibility. However, how chromatin accessibility plays a regulatory role in IMF deposition in pigs has not been reported. Xidu black is a composite pig breed with excellent meat quality, which is an ideal research object of this study. In this study, we used the assay for transposase-accessible chromatin using sequencing (ATAC-seq) and RNA sequencing (RNA-seq) analysis to identify the accessible chromatin regions and key genes affecting IMF content in Xidu black pig breed with extremely high and low IMF content. First, we identified 21,960 differential accessible chromatin peaks and 297 differentially expressed genes. The motif analysis of differential peaks revealed several potential *cis-*regulatory elements containing binding sites for transcription factors with potential roles in fat deposition, including Mef2c, CEBP, Fra1, and AP-1. Then, by integrating the ATAC-seq and RNA-seq analysis results, we found 47 genes in the extremely high IMF (IMF_H) group compared with the extremely low IMF (IMF_L) group. For these genes, we observed a significant positive correlation between the differential gene expression and differential ATAC-seq signal (*r*^2^ = 0.42). This suggests a causative relationship between chromatin remodeling and the resulting gene expression. We identified several candidate genes (*PVALB*, *THRSP*, *HOXA9*, *EEPD1*, *HOXA10*, and *PDE4B*) that might be associated with fat deposition. Through the PPI analysis, we found that *PVALB* gene was the top hub gene. In addition, some pathways that might regulate fat cell differentiation and lipid metabolism, such as the PI3K-Akt signaling pathway, MAPK signaling pathway, and calcium signaling pathway, were significantly enriched in the ATAC-seq and RNA-seq analysis. To the best of our knowledge, our study is the first to use ATAC-seq and RNA-seq to examine the mechanism of IMF deposition from a new perspective. Our results provide valuable information for understanding the regulation mechanism of IMF deposition and an important foundation for improving the quality of pork.

## Introduction

With the improvement of people’s living standards, high-quality pork is becoming increasingly popular among consumers. Intramuscular fat (IMF) is an important indicator of pork quality and is closely related to other meat quality characteristics, such as flavor, juiciness and tenderness (1). However, increasing IMF content by traditional breeding methods is extremely difficult because of the high cost of IMF trait determination (2). Therefore, understanding the genetic mechanisms affecting IMF can help improve IMF content through molecular breeding.

IMF content is a complex trait with moderate heritability (0.2–0.4) (3), and is regulated by multiple genes. In recent years, with the development of high-throughput sequencing and bioinformatics, an increasing number of studies have aimed to clarify the molecular mechanism involved in IMF trait. At present, a large number of quantitative trait loci (QTL) affecting IMF content have been identified on the basis of genome-wide association studies (GWAS). To date, 890 QTLs related to IMF content have been identified and deposited in PigQTLdb (Release 47, Apr 25, 2022^1^). Multiple candidate genes related to IMF content were identified in the pig, such as *H-FABP* (4), *PLIN1* (1), *PPAR*γ (5), *TCF7L2* (6) and *PELP1* (3). However, whether these are the key genes affecting IMF content remains ambiguous.

Transcriptome studies comparing individuals with extreme phenotypic traits are useful methods for analyzing complex traits in animals (1). For IMF content trait, several studies on transcriptome profiling in pigs have been reported. For example, RNA-seq has been used to identify differentially expressed genes associated with IMF content in the *longissimus dorsi* muscle of a single pig breed (2, 7) or two breeds (8, 9) with extreme meat quality traits. These studies have greatly improved our understanding of IMF trait. However, determining the exact biological and molecular mechanisms underlying meat quality remains a challenge, and new methods are needed to unravel the genes and pathways that regulate IMF content.

Gene expression is usually regulated by transcription factors that interact with *cis-*regulating DNA elements (10). Chromatin accessibility is an important component of epigenomics and can directly reflect the effects of chromatin structural modification on gene transcription (11). Characterizing the chromatin accessibility can help identify the regulation regions of gene expressions associated with IMF deposition and provide insights into gene regulatory mechanisms and transcriptional networks (12). Therefore, it is important to reveal differences in chromatin accessibility between groups with extremely high- and low-IMF content.

Assay for transposase-accessible chromatin with high-throughput sequencing (ATAC-seq), a valuable method of chromatin accessibility, has been widely used to discover different *cis-*regulatory elements and predict transcription factor binding sites (13–15). Recently, some researchers have studied the skeletal muscle development at different embryonic stages of Large White pigs from the perspective of chromatin opening (11). Miao et al. used ATAC-seq analysis to investigate differences in skeletal muscle growth and development between Luchuan and Duroc pigs (16). However, the molecular mechanisms underlying the differences in IMF trait have not been analyzed from the perspective of chromatin openness characteristics.

The Xidu black pig is a composite breed, with about 50% Hubei white, 25% Meishan, and 25% Enshi black pig inheritance (17). It has good meat quality, making it a good subject for this study (18). In this study, we used ATAC-seq to detect open chromatin regions and transcription factors that regulate IMF trait in Xidu black pigs with extremely high or low IMF content. By integrating the ATAC-seq and RNA-seq data, we further identified some potential key genes that regulate IMF deposition. To the best of our knowledge, our study is the first to use ATAC-seq and RNA-seq to examine the mechanism of IMF deposition in one pig breed from a new perspective. The results of this work will provide an important basis for further exploration of the molecular mechanism of IMF deposition in pigs and broaden our understanding of epigenetics during IMF deposition.

## Materials and methods

### Sample description

A total of 48 Xidu black pigs from Hubei Tianzhili high quality pig breeding Co., Ltd., (Enshi, Hubei, China) were used in this study to select groups with extremely high and low IMF content. The pigs were raised on the same commercial farm and fed the same diet under similar conditions until the average body weight reached 100 kg. The IMF contents were determined at the Breeding Swine Quality Supervision and Testing Center, Ministry of Agriculture (Wuhan, China). IMF content measurement was done by the Soxhlet petroleum method as previously described (18). *Longissimus dorsi* muscle samples were collected and frozen in liquid nitrogen for subsequent experiments. According to the IMF content, two samples of extreme high and two samples with extreme low levels were sequenced for ATAC-seq, and four samples with the highest and four samples with the lowest levels were sequenced for RNA-seq (details in Supplementary Table 1).

### ATAC-seq

A total of four samples were used to construct libraries for ATAC-seq. For all samples, raw sequence reads were initially processed for quality control by FastQC. The Fastp (v0.19.11) software was used to remove adapters and low-quality sequences to obtain high quality clean reads, and the BWA (Burrows-Wheeler Alignment) (0.7.12) software was used to compare the sequencing sequences with the reference genome (Sus scrofa 11.1). SAM files were converted to BAM format using Samtools and used for peak calling. The MACS2 (2.1.2) software was used for peak calling to obtain an overview of the open chromatin regions (-q 0.05 –call-summits –nomodel –shift -100 –extsize 200). The region would be defined as a peak when *Q* value < 0.05. The distribution of peaks in different genomic regions was assessed using ChIPseeker (v1.16.1). All sequencing tracks, bigWig files were viewed using the Integrated Genomic Viewer (IGV 2.11.7). DiffBind package in R was used to analyze peak differences across groups based on the following criteria: | log_2_(fold change)| ≥ 1 and *p* < 0.01. ATAC-seq peaks were annotated using Homer’s annotatePeaks.pl. Two biological replicates were used.

### RNA-seq

The raw reads were evaluated using FastQC (v0.11.9), and high-quality clean reads were obtained by filtering the adapter and low-quality reads using Trimmomatic (v0.39), and then mapped to the pig genome (Sus scrofa 11.1) using the Hisat2 (v2.2.1) software with the default settings. Differential gene expression analysis was performed using R package DEGseq2 between the H and L groups. Significantly differentially expressed genes (DEGs) were identified based on the following criteria: | log2(fold change)| ≥ 1 and *p* < 0.01. Four biological replicates were used.

### Gene functional annotation

Gene Ontology (GO) and Kyoto Encyclopedia of Genes and Genomes (KEGG) pathway analyses were performed by DAVID (19) (Dec. 2021). A *p*-value of 0.05 was used as the significance cutoff for GO term and pathway identification.

### Protein-protein interaction network integration and hub gene analysis

In order to analyze the connections among the proteins encoded by selected genes, Search Tool for the Retrieval of Interacting Genes (STRING^2^) database were used to search for the protein-protein interaction (PPI) pairs of selected genes with the medium confidence interaction score (0.4). The PPI file generated by STRING were imported into Cytohubba plugin app in Cytoscape 3.9.1 software with the maximal clique centrality (MCC) method to detect the top 10 hub genes.

### Validation of differentially expressed genes from transcriptome data based on qRT-pCR results

To validate the relative expressions patterns obtained by RNA-seq, qRT-PCR was performed for four individuals with extremely high IMF and four individuals with extremely low IMF contents. Total RNA was isolated using TRIzol reagent (Invitrogen, United States) and then reverse-transcribed with ABScript III RT Master Mix for qPCR with gDNA Remover (RK20429) (ABclonal, China) following the manufacturer’s instructions. cDNA was quantified using 2X Universal SYBR Green Fast qPCR Mix (RK21203) (ABclonal, China) on an ABI QuantStudio instrument (Applied Biosystems, United States). The amplification regime was conducted with 40 cycles of 95°C for 15 s, 61°C for 15 s, and 72°C for 20 s. The primers used in this study are shown in Table 1. Each gene was normalized to the housekeeping gene β*-actin*. Three biological replicates were used.

**TABLE 1:** Primers used to verify the quality of samples.

Name	Primer sequence (5′ to 3′)
CLSPN-F	AGGCTCACTGCTAAACCA
CLSPN-R	TGGACCCATTCTCCTTAC
DNAJB1-F	AGACCTCCAACAACATTCC
DNAJB1-R	AATCCTGGCTGGGTAAAT
EEPD1-F	CGAAGACGCTGGACAACA
EEPD1-R	TCCAAGGGTTCGTGAGGC
ERBB4-F	GGGAGATGACCGTATGAA
ERBB4-R	TGGGAGGTGGGATGTTGA
HOXA9-F	GCCGGACGGCAGTTGATA
HOXA9-R	TTCCAGTGTTTGGTGCTTTGT
PDE4B-F	CAAGTTCCGGTGTTCTGC
PDE4B-R	TCCATGATGCGGTCTGTC
PVALB-F	GATGCCAGAGACCTGTCAGA
PVALB-R	GAGCCTCTTAGCTTTCAGCCA
SLC38A3-F	ATCTTCCCTGCCATCTTCT
SLC38A3-R	CCCGAGACCCAGTCAATAA
THRSP-F	AGGAGGTGACGAGGAAAT
THRSP-R	CTCAGAGGAAGGGAAGGA
β-actin-F	CCAGGTCATCACCATCGG
β-actin-R	CCGTGTTGGCGTAGAGGT

## Results

### Variation in intramuscular fat content between the extreme groups

**FIGURE 1:**
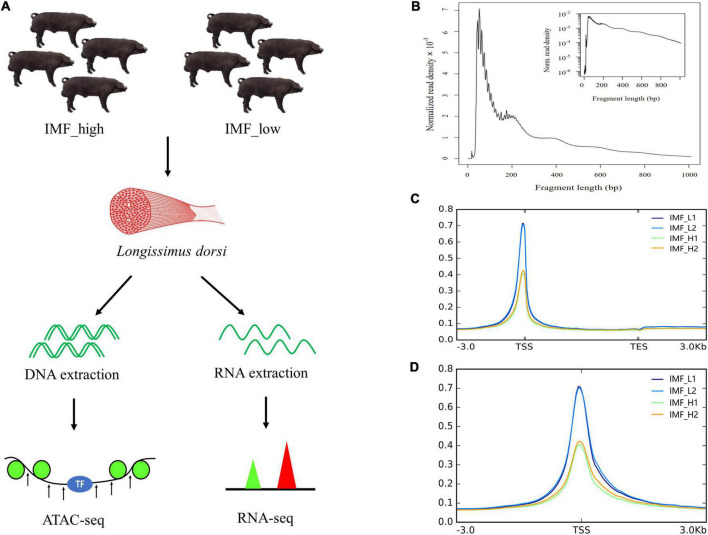
Overview of the ATAC-seq results. **(A)** Schematic representation of the experiment. *Longissimus dorsi* from the two groups (IMF_high and IMF_low groups) were sampled, and then DNA and RNA were extracted and processed for ATAC-seq and RNA-seq, respectively. **(B)** Fragment length distribution map. **(C,D)** Mapped reads distributions across gene bodies and peaks.

In this study, IMF contents in the *longissimus dorsi* muscle of 48 Xidu black pigs were measured to select samples for ATAC-seq and RNA-seq. The IMF content varies among individuals, ranging from 1.16 to 5.28%. On the basis of the IMF content, four individuals with the highest IMF were divided into the high group (IMF_H) and the 4 individuals with the lowest IMF into the low group (IMF_L) (Supplementary Table 1). The mean IMF content in the two group was 4.95 ± 0.49 and 1.30 ± 0.12, respectively. After statistical analysis, the difference of IMF content between the two groups was statistically significant (*p* < 0.01), while carcass weight did not differ significantly between the two groups. Here, all eight samples were subjected to RNA-seq analysis, and four samples were subjected to ATAC-seq analysis (Figure 1A).

### ATAC-seq quality control of the Xidu black pig muscle tissues

To investigate the genome-wide accessible chromatin regions involved in IMF content, we profiled the accessibility of chromatin between the higher and lower IMF content of pigs by using ATAC-seq. A total of 268,792,325 raw reads were obtained. After filtration, 263,561,596 clean reads were uniquely mapped to the reference genome (Table 2). We first assessed the quality of the libraries on the basis of the lengths of the inserts and peak signal distributions. All ATAC-seq libraries showed the expected, including one nucleosome-free fragment and one mononucleosome fragment, indicating good data quality (Figure 1B). For the distribution of fragment length, many previous ATAC-seq data also show a similar phenomenon (20–22). The results of mapped reads distributions across the gene bodies and peaks also showed good quality of the ATAC-seq (Figures 1C,D). Most of the identified accessible areas were enriched in the 3 kb of transcription start site (TSS), indicating that open regions of chromatin were involved in transcriptional regulation. To further confirm the quality of ATAC-seq, principal component analysis (PCA) was performed on all samples. The results showed a similarity between the biological replicates and a difference between the two groups (Supplementary Figure 1). These results suggest that the quality of the sequencing data was very high.

**TABLE 2:** Summary of the ATAC-seq data.

Sample	Raw reads	Raw bases (G)	Raw Q30	Clean reads	Clean bases (G)	Clean base rate	Clean Q30
IMF_H1	64,179,061	19.25	0.88	62,847,347	15.08	0.78	0.92
IMF_H2	86,623,835	25.99	0.88	85,097,723	22.55	0.87	0.91
IMF_L1	54,820,002	16.45	0.84	53,834,792	12.85	0.78	0.89
IMF_L2	63,169,427	18.95	0.87	61,781,734	15.15	0.80	0.91
Total	268,792,325	80.64		263,561,596	65.63	3.23	

### Genome-wide identification of accessible chromatin regions

**FIGURE 2:**
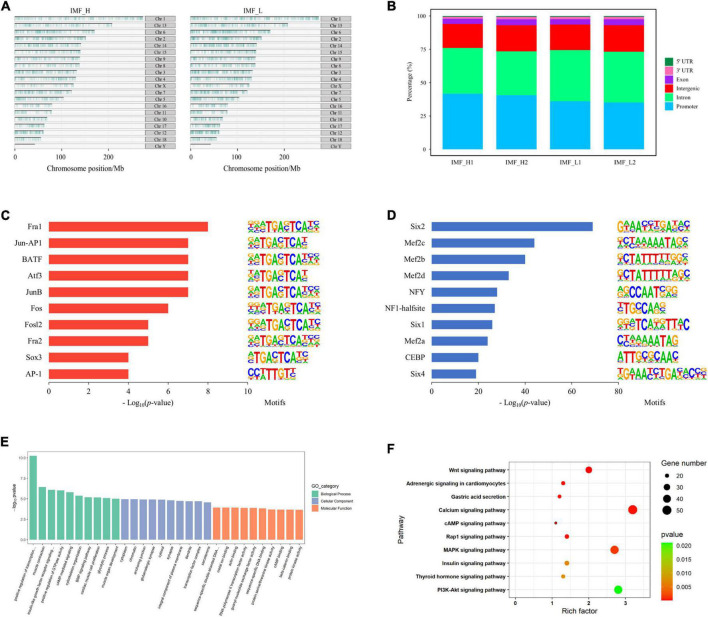
Distribution of peaks and analysis of differential peaks. **(A)** The distribution of peaks on the chromosomes. **(B)** Genomic distribution of the peaks in each sample. Genomic functional regions include promoter, intergenic, exon, intron, 5’UTR and 3’UTR. **(C)** Enriched transcription factor binding motifs by increased peaks between the IMF_H and IMF_L by ATAC-seq. **(D)** Enriched transcription factor binding motifs by decreased peaks. **(E)** GO enrichment analysis of genes corresponding to differential peaks. **(F)** KEGG pathway enrichment analysis of genes corresponding to differential peaks.

We identified 111,283 and 141,423 accessible chromatin peaks in IMF_H and IMF_L groups, respectively. The distribution of peaks on the chromosomes of the pig genome is shown in Figure 2A. It can be seen that most regions of each chromosome were covered, and some chromosomes such as ChrX and ChrY were less covered. Using the annotation file, we annotated the genomic distribution of open chromatin peaks across all samples. As expected, most peaks mapped to the promoter, intronic, and intergenic regions (Figure 2B).

To identify the open sites related to IMF traits, we compared differences in the identified peaks between the IMF_H and IMF_L by ATAC-seq. There were 5,367 up-regulated (more accessible chromatine) and 16,593 down-regulated (less accessible chromatine) peaks in the IMF_H vs IMF_L group. The top 10 significantly enriched transcription factor binding motifs by increased peaks were known as the binding sites of Six, Mef2c, NF1-halfsite and CEBP (Figure 2C), while those enriched by decreased peaks contained binding sites for Fra1, Jun-AP1, JunB and AP-1 (Figure 2D).

By annotating each different peak, the IMF_H group showed 1,817 down-regulated genes and 611 up-regulated genes (Supplementary Table 2), compared with the IMF_L group. GO and KEGG pathway enrichment analysis were performed for these genes. All GO terms were summarized into three main GO categories, biological process, cellular component and molecular function. The top 10 GO terms of each categories were shown in Figure 2E. We found that the GO analyses were primarily enriched in muscle contraction, glycolytic process, muscle organ development and transcription factor complex. Moreover, the top 10 significantly enriched KEGG pathways were shown in Figure 2F, many of which were involved in lipid metabolism and adipocyte differentiation, such as PI3K-Akt signaling pathway (23), MAPK signaling pathway (24), calcium signaling pathway (25).

### RNA-seq data

To detect the gene expression patterns in the IMF_H and IMF_L groups, we selected the four samples with the highest IMF contents and the four samples with the lowest IMF contents for RNA-seq analysis. A total of 960 million reads were obtained from the transcriptome sequencing of the eight samples. After trimming and filtering, 940 million reads remained. Between 92.5% and 93.8% of the reads were mapped against the pig reference genome across the samples (Table 3). In total, 17,401 and 17,307 coding genes were identified in the IMF_H and IMF_L groups, respectively. Of these, 16,094 genes were shared between the two groups. The PCA result showed that the four IMF_H samples clustered together, which was significantly different from the clusters of the four IMF_H samples (Supplementary Figure 2).

**TABLE 3:** Summary of the RNA-seq data.

Sample	Raw reads (M)	Raw bases (G)	Clean reads (M)	Clean bases (G)	Q30 (%)	Unique mapped reads
IMF_H1	119.94	11.48	114.80	11.47	90.96	106,229,092 (92.54%)
IMF_H2	117.44	11.34	113.43	11.33	91.8	105,870,112 (93.34%)
IMF_H3	119.94	11.56	115.64	11.55	92.23	107,097,048 (92.61%)
IMF_H4	122.44	12.43	124.29	12.42	94.54	115,657,413 (93.06%)
IMF_L1	119.94	11.58	115.81	11.57	92.22	107,174,114 (92.54%)
IMF_L2	122.44	11.80	117.97	11.78	91.34	110,680,739 (93.82%)
IMF_L3	117.44	11.32	113.18	11.31	91.57	104,900,706 (92.69%)
IMF_L4	119.94	12.42	124.20	12.41	94.58	114,907,722 (92.52%)

**FIGURE 3:**
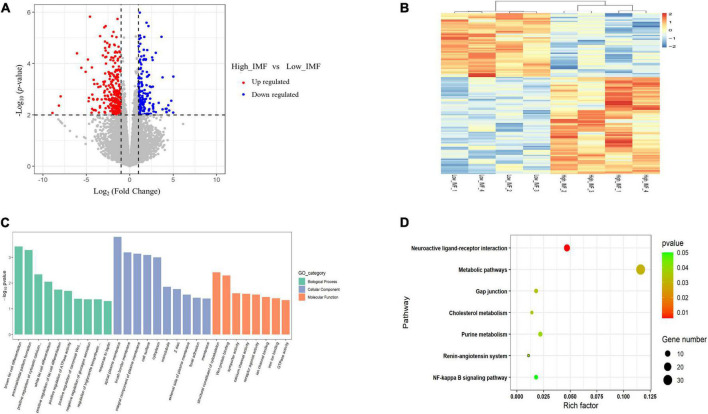
Analyses of RNA-seq. **(A)** Scatter plot of the transcriptome data [*p* < 0.05, | log2(fold change)| ≥ 1]. **(B)** Heatmap of the differentially expressed genes. **(C)** GO enrichment analysis of differentially expressed genes. **(D)** Bubble chart of Kyoto Encyclopedia of Genes and Genomes (KEGG) pathway enrichment analysis of differentially expressed genes.

To identify key functional genes affecting IMF content, differentially expressed genes (DEGs) were filtered based on the criteria | log_2_(fold change)| ≥ 1 and *p* < 0.01. Overall, 297 DEGs were identified, including 222 upregulated and 75 downregulated genes (Figure 3A). The heat maps of these DEGs are shown in Figure 3B, from which it can be seen that the expression patterns of DEGs are consistent within groups, but different between groups. Detailed information for all DEGs is shown in Supplementary Table 3. GO term and KEGG pathway enrichment analysis were used to investigate the potential functions of DEGs in the regulation process of IMF. We found that the biological process of GO analysis were primarily enriched in brown fat cell differentiation, white fat cell differentiation and response to leptin, etc., whereas cell component were mainly enriched in Wnt-protein binding, calcium channel activity and GTPase activity (Figure 3C). In addition, KEGG analysis showed that the DEGs were enriched in pathways related to metabolism, including purine metabolism, cholesterol metabolism and metabolic pathways (Figure 3D). This suggested that those genes may be involved in fat cell differentiation and muscle development.

### Integration of the ATAC-seq results with RNA-seq

**FIGURE 4:**
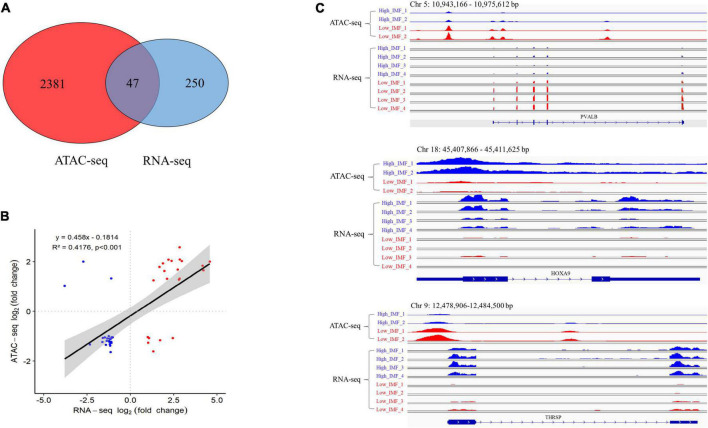
Integration analysis of ATAC-seq and RNA-seq results. **(A)** Overlap of differentially expressed genes identified by ATAC-seq and RNA-seq. **(B)** Correlation of significantly differentially accessible gene (ATAC-seq) and gene expression (RNA-seq). **(C)** IGV snapshot for ATAC-seq and RNA-seq signal for the *PVALB*, *HOXA9* and *THRSP* gene.

To investigate whether the changes in open chromatin regions were correlated with the changes in gene expression levels, we performed an integrative analysis of the ATAC-seq and RNA-seq data sets. A total of 47 overlapping genes were identified (Supplementary Table 4 and Figure 4A), including 19 upregulated and 28 downregulated genes in ATAC-seq, 22 upregulated and 25 downregulated genes in RNA-seq. We did a correlation analysis of the expression levels and chromatin openness of these 47 overlapping genes and observed a significant positive correlation between the differential gene expression and differential ATAC-seq signal (*r*^2^ = 0.42; Figure 4B). Some examples are shown in Figure 4C. The chromatin accessibility and transcription level of *HOXA9* (homeobox A9) in IMF_H were higher than those in IMF_L. On the contrary, the chromatin accessibility and transcription level of *PVALB* (parvalbumin) in IMF_L were higher than those in IMF_H. Interestingly, the chromatin accessibility of *THRSP* (thyroid hormone responsive) in IMF_H was higher than that in IMF_L, however, the transcription level was lower.

### STRING protein-protein interaction network analysis

**FIGURE 5:**
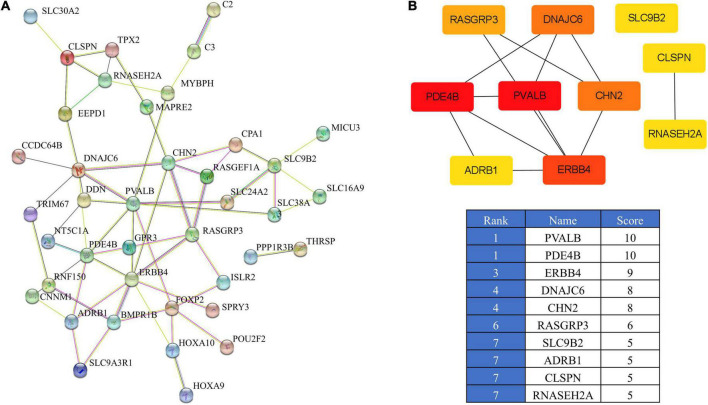
PPI network of the overlapping genes. **(A)** PPI analysis of 47 overlapping genes from IMF_H vs. IMF_L comparison according to the STRING database. **(B)** PPI network of the top 10 hub genes visualized using Cytoscape.

To further identify the core genes, we established a PPI network of the 47 overlapping genes using STRING (Figure 5A). This information was then imported into the cytohubba in Cytoscape to identify the top 10 hub genes (Figure 5B). The network contained 46 nodes and 61 edges. Among the 46 nodes, the top 10 most strongly connected PPI nodes were selected as hub genes. These hub genes were *PVALB*, *PDE4B*, *ERBB4*, *DNAJC6*, *CHN2*, *RASGRP3*, *SLC9B2*, *ADRB1*, *CLSPN*, and *RNASEH2A*.

### Validation of the results by qRT-pCR

**FIGURE 6:**
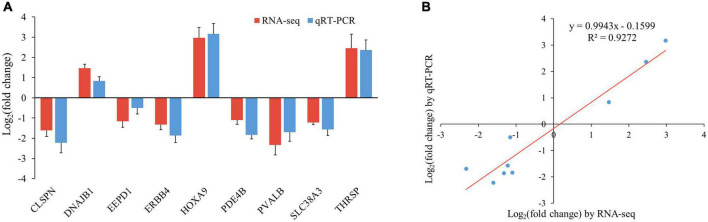
Verification of RNA-seq data by qRT-PCR. **(A)** Histogram of RNA-seq and qRT-PCR expression levels. X-axis represents 9 selected genes, and Y-axis represents the expression levels of genes from RNA-seq and qRT-PCR. **(B)** The liner regression analysis of expression level between RNA-seq and qRT-PCR data. The X-axis represents the log_2_ fold change of RNA-seq, and the Y-axis indicates the log_2_ fold change of qRT-PCR.

To validate the accuracy of the RNA-seq data, nine genes randomly selected from the top 20 DEGs (*CLSPN*, *DNAJB1*, *EEPD1*, *ERBB4*, *HOXA9*, *PDE4B*, *PVALB*, *SLC38A3*, and *THRSP*) were analyzed by qRT-PCR. The results showed that the expression pattern of these genes from qRT-PCR was consistent with RNA-seq (Figure 6A), and the correlation between the two methods was relatively high, with a correlation coefficient of 0.96 (*r*^2^ = 0.93, Figure 6B), indicating that DEGs identified from RNA-Seq in this study were reliable.

## Discussion

IMF content has an important effect on the quality and flavor of pork, and increasing IMF content has become an important goal in modern pig breeding. The identification of key genes affecting IMF is useful in molecular breeding to increase IMF content. In recent years, several research groups have studied the mechanism of IMF formation, providing basic information for further research (1, 2, 18). As the regulatory mechanisms affecting IMF formation are complex and not well studied, it is important to examine biological issues at different levels. Therefore, the integration analysis of multi-omics can further identify the key factors of a biological process, as well as the target genes of a transcription factor.

In the present study, we detected, for the first time, the changes in chromatin accessibility and gene expression in the *longissimus dorsi* muscle of one pig breed with extremely high and low IMF content to identify the key factors involved in IMF deposition. First we comprehensively analyzed the changes in chromatin accessibility between the two groups. ATAC-seq identified 21,960 down-regulated and 5,367 up-regulated peaks in the IMF_H group compared with the IMF_L group. Among the predicted transcription factors that contained binding sites in the increased or decreased peaks, several transcription factors were involved in adipocyte differentiation and meat quality, such as Mef2c, CEBP, Fra1, and AP-1. Mef2c, as a transcription factor, has been well studied in the specificity of the muscle fiber type, but recent reports have begun to reveal its role in adipogenesis (26). In pigs, *Myostatin* could regulate fatty acid desaturation and fat deposition through Mef2c/miR222/SCD5 cascade (27). Fra1 has been shown to affect aging-related intramuscular adipose tissue in the aged skeletal muscles of mice through FGF-2/FRA-1/miR-29a/SPARC axis (28). The Fos-related antigen 2 (Fra-2) and activator protein-1 (AP-1) transcription factors are important regulators of adipocyte differentiation. Fra-2/AP-1 could control adipocyte differentiation and survival by regulating PPARc and hypoxia (29). The CCAAT/enhancer binding protein (CEBP) family, is a subfamily of the basic leucine zipper (bZIP) transcription factor superfamily and regulates many important biological processes, such as energy metabolism, inflammation, adipose proliferation and differentiation (30, 31). The CEBP family was found to promote the differentiation of 3T3-L1 adipocyte (32).

The integration of the ATAC-seq and RNA-seq results showed 47 genes in the IMF_H group compared with the IMF_L group. We focused on the 10 genes with the highest *p*-values in the transcriptome. Ten of these genes, including *PVALB*, *THRSP* (33, 34), *HOXA9* (35), *EEPD1* (36), *HOXA10* (37), and *PDE4B* (38) have been reported to be involved in fat deposition and meat quality. Through the PPI analysis, we found that *PVALB* gene was the top 1 hub gene. The *PVALB* gene was closely associated with lipid and energy metabolism. Previous genome-wide transcriptome analysis of *longissimus dorsi* muscle of Laiwu pigs at four different developmental stages, had already indicated that the *PVALB* gene may be related to IMF deposition (39). Other studies have also shown that the *PVALB* gene expression levels are significantly different in pig breeds differing in fat and meat quality traits (16, 40), suggesting that the *PVALB* gene might be an important target gene for improving IMF in our studied pig. Furthermore, through the GO term and KEGG pathway analyses revealed some classical pathways involved in the regulation of fat cell differentiation and lipid metabolism, such as brown fat cell differentiation, white fat cell differentiation and response to leptin, PI3K-Akt signaling pathway, MAPK signaling pathway, calcium signaling pathway. Interestingly, several studies have demonstrated that the PI3K/Akt pathway is related to adipocyte differentiation. GPR39 can activate the proliferation and differentiation of porcine intramuscular preadipocytes by targeting the PI3K/AKT cell signaling pathway (23). CTRP6 can regulate the proliferation and differentiation of porcine adipocytes through AdipoR1/MAPK signaling pathway (41). CRTC3 can regulate lipid metabolism and adipogenesis in the intramuscular and subcutaneous adipocytes of pigs by activating calcium channels (25), further validating the biological importance of genes in the GO terms and KEGG pathways reported in our study especially as it relates to IMF deposition.

## Conclusion

Taken together, this study provided a novel resource for identifying open chromatin regions and transcription factors involved in the regulation of IMF deposition in Xidu black pigs with extremely high and low IMF traits. By integrating ATAC-seq and RNA-seq analyses, we identified several potential candidate genes related to fat deposition, such as *PVALB*, *THRSP*, *HOXA9*, *EEPD1*, *HOXA10* and *PDE4B*, and pathways such as PI3K-Akt signaling pathway, MAPK signaling pathway, calcium signaling pathway. These potential candidate genes and pathways play important roles in understanding IMF deposition in pigs, and the specific mechanisms of their effect on IMF content warrant further study. To our knowledge, this is the first study to focus on IMF trait using ATAC-seq combined with RNA-seq analysis. These findings provide valuable information for understanding the regulatory mechanism of IMF deposition and conducting meat quality genetic improvement projects.

## Data availability statement

The datasets presented in this study can be found in online repositories. The names of the repository/repositories and accession number(s) can be found at: http://doi.org/10.6084/m9.figshare.20390538.

## Ethics statement

All experimental procedures were approved by the Institutional Animal Care and Use Committee of the Hubei Academy of Agriculture Sciences, and all methods that involved pigs were in accordance with the agreement of the Institutional Animal Care and Use Committee of the Hubei Academy of Agriculture Sciences (permit number: 36/2016).

## Author contributions

XP, SM, and JW designed the study. ZX, JZ, YZ, and MQ performed the data collection. ZX performed the analyses under the assistance and guidance of HS, ZL, LL, NC, and FO. ZX drafted the manuscript. All authors read and approved the final manuscript.

## Footnotes

1
https://www.animalgenome.org/cgi-bin/QTLdb/SS/index

2
https://string-db.org/
