# Supplementary material for: Integration of ATAC-seq and RNA-seq analysis identifies key genes affecting intramuscular fat content in pigs
Source: Front Nutr. 2022 Oct 5;9:1016956. doi: 10.3389/fnut.2022.1016956 (PMC9581296; doi:10.3389/fnut.2022.1016956)
Supplement: Supplementary file 1 [file Data_Sheet_1.pdf]

Integration of ATAC-seq and RNA-seq analysis identifies key genes affecting intramuscular fat content in pigs

Zhong Xu[1§], Junjing Wu[1§], Jiawei Zhou[1], Yu Zhang[1], Mu Qiao[1], Hua Sun[1], Zipeng Li[1], Lianghua Li[1], Nanqi Chen[1], Favour Oluwapelumi Oyelami[2], Xianwen Peng[1*], Shuqi Mei[1*]

[1]Hubei Key Laboratory of Animal Embryo and Molecular Breeding, Institute of Animal Husbandry and Veterinary, Hubei Provincial Academy of Agricultural Sciences, Wuhan 430064, China

[2]The John Curtin School of Medical Research, Australian National University, Canberra, Australia

[§]These authors contributed equally to this work and share first authorship.

Running title: ATAC-seq and RNA-seq of Pig IMF

*** Corresponding author:**

Xianwen Peng

pxwpal@163.com;

Shuqi Mei

msqpaper@163.com.

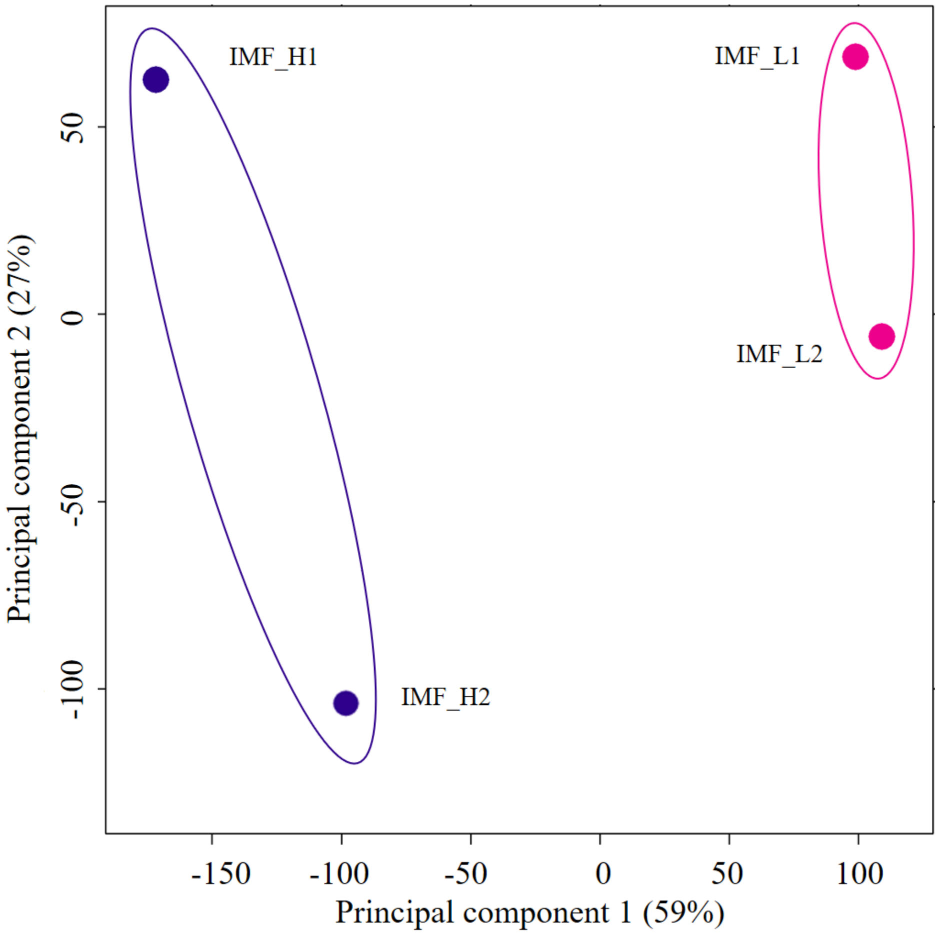

Supplementary Figure 1. The principal component analysis of ATAC-seq.

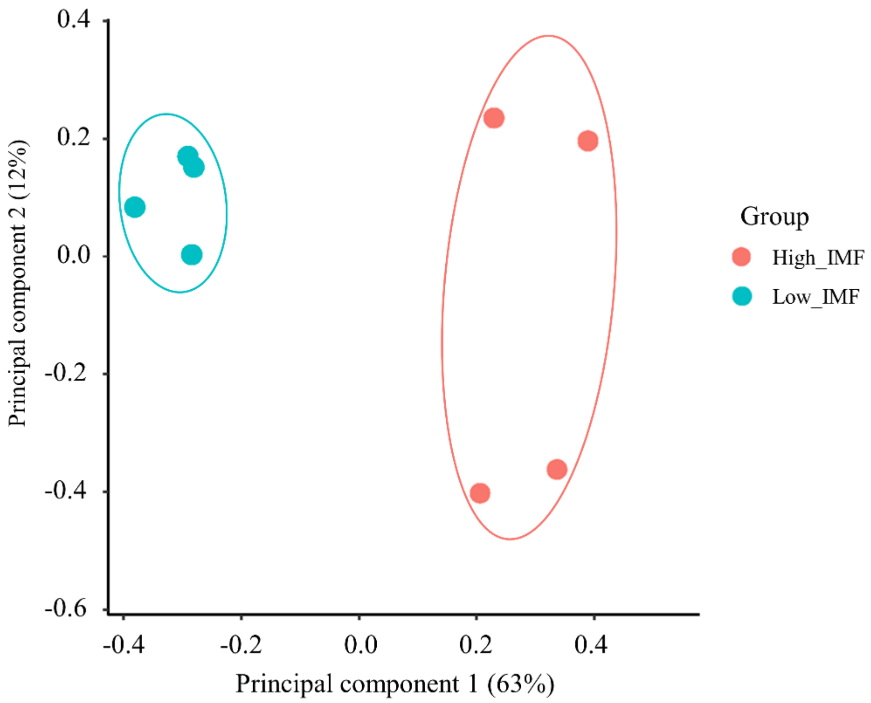

Supplementary Figure 2. The principal component analysis of RNA-seq.
